# Overexpression of the Soybean NAC Gene *GmNAC109* Increases Lateral Root Formation and Abiotic Stress Tolerance in Transgenic *Arabidopsis* Plants

**DOI:** 10.3389/fpls.2019.01036

**Published:** 2019-08-16

**Authors:** Xuefei Yang, Moon Young Kim, Jungmin Ha, Suk-Ha Lee

**Affiliations:** ^1^Department of Plant Science and Research Institute of Agriculture and Life Sciences, Seoul National University, Seoul, South Korea; ^2^Plant Genomics and Breeding Institute, Seoul National University, Seoul, South Korea

**Keywords:** abiotic stress tolerance, soybean, NAC, transcription factor, lateral roots, overexpression

## Abstract

NACs are plant-specific transcription factors that have crucial roles in plant development and biotic and/or abiotic stress responses. This study characterized the functions of the soybean NAC gene *GmNAC109* using an overexpression construct in *Arabidopsis* lines. Sequence analysis revealed that *GmNAC109* is highly homologous to *ATAF1* (*Arabidopsis Transcription Activation Factor 1*), which regulates biotic and abiotic stress responses. GmNAC109 protein localized to the nucleus and its C-terminal domain exhibited transcriptional activation activity. Salt, dehydration, and cold stresses significantly increased expression of *GmNAC109* in soybean. Similarly, *Arabidopsis* plants overexpressing *GmNAC109* were more tolerant to drought and salt stress than wild-type Col-0 plants. Stress response-related genes, such as *DREB1A* (*drought-responsive element-binding 1A*), *DREB2A*, *AREB1* (*ABSCISIC ACID-RESPONSIVE ELEMENT BINDING PROTEIN 1*), *AREB2*, *RD29A* (*RESPONSIVE TO Desiccation 29A*), and *COR15A* (*COLD REGULATED 15A*) were upregulated in *GmNAC109*-overexpressing transgenic *Arabidopsis* lines. The transgenic lines showed upregulation of the ABA-responsive genes *ABI1* (*ABA INSENSITIVE 1*) and *ABI5* and hypersensitivity to ABA. However, *GmNAC109* did not increase expression of the ABA-biosynthetic gene *NCED3* (*NINE-CIS-EPOXYCAROTENOID DIOXYGENASE* 3) and endogenous ABA content in the transgenic lines. Overexpression of *GmNAC109* significantly increased lateral root formation in transgenic *Arabidopsis* lines. Expression of *AIR3* (*AUXIN-INDUCED IN ROOT CULTURES 3*) and *ARF2* (*AUXIN RESPONSE FACTOR 2*) was increased and decreased in these transgenic lines, respectively, indicating that *GmNAC109* is involved in the auxin signaling pathway and thereby helps to regulate hairy root formation. Our results provide a basis for development of soybean lines with improved tolerance to abiotic stresses *via* genetic manipulation.

## Introduction

Soybean (*Glycine max* [L.] Merr.) is one of the most important crops for human food/livestock feed and has diverse uses in industrial products due to its high protein and oil contents. Worldwide soybean production was estimated at 338.6 million tons in 2018 (FAO, 2019). However, biotic and abiotic stresses affect soybean yield, with drought stress having the greatest effect. Water deficit during reproductive growth can cause flower abscission, abortion of embryos, and a reduced pod number, resulting in production of fewer and smaller seeds (Andriani et al., 1991).

NACs form an important family of plant transcription factors (TFs) and are associated with various developmental processes, including apical shoot development (SAM) (Aida et al., 1999), leaf senescence (Guo and Gan, 2006), and secondary wall formation (Mitsuda et al., 2005), as well as responses to abiotic/biotic stresses (Nuruzzaman et al., 2013). Members of this gene family contain a NAC domain (*Petunia* NAM *Arabidopsis* ATAF1/2, and CUC2) (Souer et al., 1996; Aida et al., 1997). Genome sequencing identified 151 NAC family members in *Oryza sativa* and 126 in *Arabidopsis thaliana* (Shao et al., 2015). In *Arabidopsis*, some NACs have been reported to play an important role in abiotic stress responses. For example, oxidative and abiotic stresses such as high light, osmolarity, and salinity induce expression of *ANAC032* (*Abscisic-acid-responsive NAC*), leading to repression of anthocyanin biosynthesis and positive regulation of leaf senescence (Mahmood et al., 2016a; Mahmood et al., 2016b). Overexpression of other NAC genes, *ANAC019*, *ANAC055*, and *ANAC072*, significantly enhances drought tolerance (Li et al., 2012). Various abiotic signals also induce expression of *ATAF1*, a member of the NAC family in *Arabidopsis*, and overexpression of this gene causes dwarfism and short primary roots (Wu et al., 2009). In rice, overexpression of *ATAF1* enhances tolerance to salt and sensitivity to abscisic acid (ABA) (Liu et al., 2016).

Many studies have investigated the functions of *GmNAC* genes since NAC genes were first cloned in soybean (Meng et al., 2007). *GmNAC30* and *GmNAC81* regulate the stress-induced programmed cell death response (Mendes et al., 2013). *GmNAC003*, *GmNAC004*, *GmNAC11*, and *GmNAC20* function in the abiotic stress response and affect lateral root development (Hao et al., 2011; Quach et al., 2014). Genome-wide analysis identified at least 180 members of the soybean *GmNAC* superfamily (Melo et al., 2018). Expression levels of several *GmNAC* genes, such as *GmNAC019*, *GmNAC109*, and *GmNAC148*, have been measured in different tissues, including roots, flowers, leaves, and pods, of drought-resistant and drought-sensitive soybean cultivars under drought stress. These genes were found to be possible candidates for drought tolerance (Le et al., 2011; Hussain et al., 2017). The functions of only a few members of the soybean NAC family have been studied to date, and the functions of more *GmNAC* genes must be investigated to comprehensively understand their specific and redundant roles in stress tolerance.

We selected *GmNAC109* (Glyma_14G152700) among three genes (*GmNAC019*, *GmNAC109*, and *GmNAC148*) that were previously reported to be candidates for drought stress resistance based on transcriptomic profiling (Le et al., 2011; Hussain et al., 2017). To verify the function of *GmNAC109*, we constructed *Arabidopsis* transgenic lines that overexpressed this gene and compared their morphology and stress tolerance with those of wild-type plants. Additionally, expression levels of other stress-responsive TFs and ABA-related genes were analyzed in the *GmNAC109*-overexpressing lines.

## Materials and Methods

### Plant Materials and Growth Conditions

The soybean cultivar Williams 82 was used in RT-PCR and quantitative real-time PCR experiments. *A. thaliana* ecotype Columbia (Col-0) was used for the β-Glucuronidase assay and *Agrobacterium*-mediated floral dip transformation. Tobacco (*Nicotiana benthamiana*) was used for subcellular localization analysis of *GmNAC109 via* transient expression assay. Seedlings of soybean, *Arabidopsis*, and tobacco were all grown at 25°C under a 16/8-h light/dark cycle at 60% humidity.

### Soybean Abiotic Stress Treatments

For soybean abiotic stress treatments, 14-day-old Williams 82 seedlings were removed from soil, and the remaining soil was carefully washed away with flowing water. For salt treatment, the seedling roots were soaked in 150 mM NaCl solution for 0 (as a control), 2, 5, and 10 h. For dehydration stress treatment, roots were wiped carefully and kept under the same growth conditions, but with 25% humidity for 0 (as a control), 2, 5, and 10 h. For cold stress treatment, seedling roots were soaked in 1/2 Murashige and Skoog (MS) liquid medium and kept at 4°C for 0 (as a control), 2, 5, and 10 h. Shoot, leaf, and root tissues were collected from seedlings exposed to these three stress treatments. Each experimental set had three biological replicates, and each replicate contained three plants. Significant differences between groups were analyzed by Student’s *t* test.

### Quantitative Real-Time PCR (qRT-PCR)

To examine *GmNAC109* expression in soybean under different stresses and stress-related genes in *Arabidopsis* transgenic lines, total RNA was extracted using a GeneAll Ribospin™ Plant Kit (Cat. 307-150; GeneAll, Seoul, Korea). Approximately 1 μg of total RNA was reverse-transcribed using an iScript™ cDNA Synthesis Kit (Cat. 1708891; Bio-Rad, CA, USA). qRT-PCR was performed using iQ^™^ SYBR^®^Green Supermix (Cat. 170-8880AP; Bio-Rad). PCR was conducted on a LightCycler 480 Real-Time PCR system (Roche Diagnostics, Laval, QC, Canada). *ACTIN11* and *EF-1α* (AT5G60390) were used as reference genes for soybean and *Arabidopsis* samples, respectively (Jian et al., 2008). All primers were designed by primer3 (http://bioinfo.ut.ee/primer3-0.4.0/) (**Supplementary Table S1**). Each sample has three biological replicates, and each biological replicate has two technical replicates. The significant difference between groups was analyzed by Student’s *t* test.

### β-Glucuronidase Assay

The promoter region of *GmNAC109*, a 2-kb fragment upstream from the start codon, was amplified with primers that included a 25-bp *attB1* site (**Supplementary Table S1**). PCR products were cloned into the pDONR221 vector by the BP recombination reaction, and the sequence was confirmed by Sanger sequencing. The promoter sequence was then cloned upstream of the β-glucuronidase (GUS) gene in the pGWB633 vector (Nakamura et al., 2010) by the LR recombination reaction. The *Arabidopsis* Col-0 line was transformed with the resulting construct by the floral dip method (Zhang et al., 2006). GUS staining assays of T1 transgenic *Arabidopsis* plants were performed as described by Jefferson et al. (1987). The experimental tissues were soaked in X-Gluc staining solution at 37°C overnight and washed with 95% ethanol.

### Subcellular Localization Analysis

The full-length coding sequence (CDS) of *GmNAC109*, excluding the stop codon, was amplified with primers containing 25-bp *attb* sites (**Supplementary Table S1**). The PCR product was cloned into the pDONR221 vector, and the sequence was confirmed by Sanger sequencing. The verified sequence was then inserted into the pEG101 destination vector in frame with yellow fluorescent protein (YFP) at the C-terminus. The empty pEG101 vector containing only the YFP sequence was used as a control. Four-week-old tobacco leaves were transiently transformed with the empty and GmNAC109-YFP vectors by *Agrobacterium*-mediated methods (Wydro et al., 2006). The *Agrobacterium* strain EHA105 was used. Fluorescence was checked at 48 h after agroinfiltration using an SP8X confocal laser scanning microscope (Leica, Wetzlar, Germany) with an argon laser. Excitation wavelength was 514 nm and wavelength range of captured light was 520–585 nm.

### Transactivation Assay

The full-length *GmNAC109* CDS as well as N-terminal (1–399 bp) and C-terminal (400–843 bp) fragments were amplified by PCR. The forward and reverse primers included the *Nco*I restriction sequence “CCATGG” and the *Eco*RI restriction sequence “GAATTC”, respectively. PCR products were digested with these two restriction enzymes and ligated into the pGBKT7 vector (Clontech, California, USA). The construct was sequenced and transformed into the yeast strain AH109 with the Yeastmaker^TM^ Yeast Transformation System II (Cat. 630439, Clontech). Yeast cells were spread on SD/-Trp medium to screen for positive transformants. SD/-His medium was used to examine transactivation activity. The colony lift colorimetric assay for β-galactosidase activity was carried out as described previously (Möckli and Auerbach, 2004). Yeast colonies were plated on Yeast Peptone Dextrose Adenine. Two pieces of filter paper were placed in an empty Petri dish and completely soaked in 2 ml of Z-buffer containing 11 μl of β-mercaptoethanol and 100 μl of 4% (w/v) X-gal. The upper filter paper was placed onto the yeast colony and transferred to a liquid nitrogen bath for 10 s. With the yeast colony on the top side, the filter paper was placed back onto the remaining filter paper in the Petri dish. The Petri dish containing the filter papers was incubated at 37°C.

### Construction of the *GmNAC109* Overexpression Vector and *Arabidopsis* Floral Dip Transformation

The full-length CDS of *GmNAC109* was cloned into the pENTR^™^/D-TOPO^®^ vector, and the sequence was confirmed by Sanger sequencing. This sequence was then cloned downstream of the CaMV 35S promoter in the pGWB602 vector (Nakamura et al., 2010) by the LR recombination reaction. The *A. thaliana* Col-0 line was transformed with the construct using the floral dip method and the *Agrobacterium* strain GV3101 (Zhang et al., 2006). T1 seeds were germinated on soil and sprayed with 1/500 (v/v) Basta every 3 days to screen for positive transgenic lines. T3 transgenic lines were subjected to further molecular and phenotypic analyses.

### ABA Sensitivity Assay and Stress Treatment of Transgenic *Arabidopsis* Plants

For ABA sensitivity assay, 100 seeds from Col-0 and transgenic lines were surface sterilized and sown onto 1/2 MS plate containing 1 μM ABA and stored at 4°C for 2 days in the darkness for vernalization. Germination rates were scored every 24 h for 5 days after vernalization and the photographs were taken on the 5th day.

For *Arabidopsis* abiotic stress treatments, Col-0 and transgenic lines were germinated on soil and transgenic lines were sprayed with 1/500 (v/v) Basta (Byer, Leverkusen, Germany) every 3 days. One-week-old seedlings were transferred to the pot containing equal amount of soil. For drought treatment, the pots were soaked in water for 10 min to absorb enough water and placed in the growth chamber without watering. Ten days later, re-water the pots and check the survival rate the next day. For salt treatment, the pots were watered with 250 mM NaCl solution every other day and the survival rate was examined after 10 days of treatment.

To check seed germination rate to salt stress, 100 seeds of Col-0 and transgenic lines were surface sterilized and sown on 1/2 MS medium containing 150 mM NaCl. The number of germinated seeds was counted 7 days after sowing.

All the statistical significance differences between groups were analyzed by Student’s *t* test based on three independent experiments.

### ABA Extraction and Measurement

One-week-old seedlings of Col-0 and *Arabidopsis* transgenic lines were transplanted on 1/2 MS medium with or without 75 mM Mannitol. After 1 week growth, the seedlings were collected to measure ABA contents. ABA was extracted as previously described (Yang et al., 2001). The seedlings were ground in liquid nitrogen and 100-mg sample was collected in 10 ml of extraction buffer (80% methanol containing 1 mM butylated hydroxytoluene). The extract was incubated at 4°C in darkness overnight and centrifuged at 12,000 rpm at 4°C for 20 min. To avoid the effects of plant pigment and other nonpolar compound on immunoassay, the supernatant was passed through C18 columns (C18 Sep-Park Cartridge, Waters Corp., Milford, USA). The extract eluted with 100% methanol was dried with a vacuum freeze dryer and dissolved in 500 μl of TBS buffer containing 10% methanol. The ABA content was quantified by ELISA method (Phytodetek^®^ Immunoassay kit, Agdia, Inc., Elkhart, USA).

## Results

### Tissue-Specific Expression of *GmNAC109*

**Figure 1:**
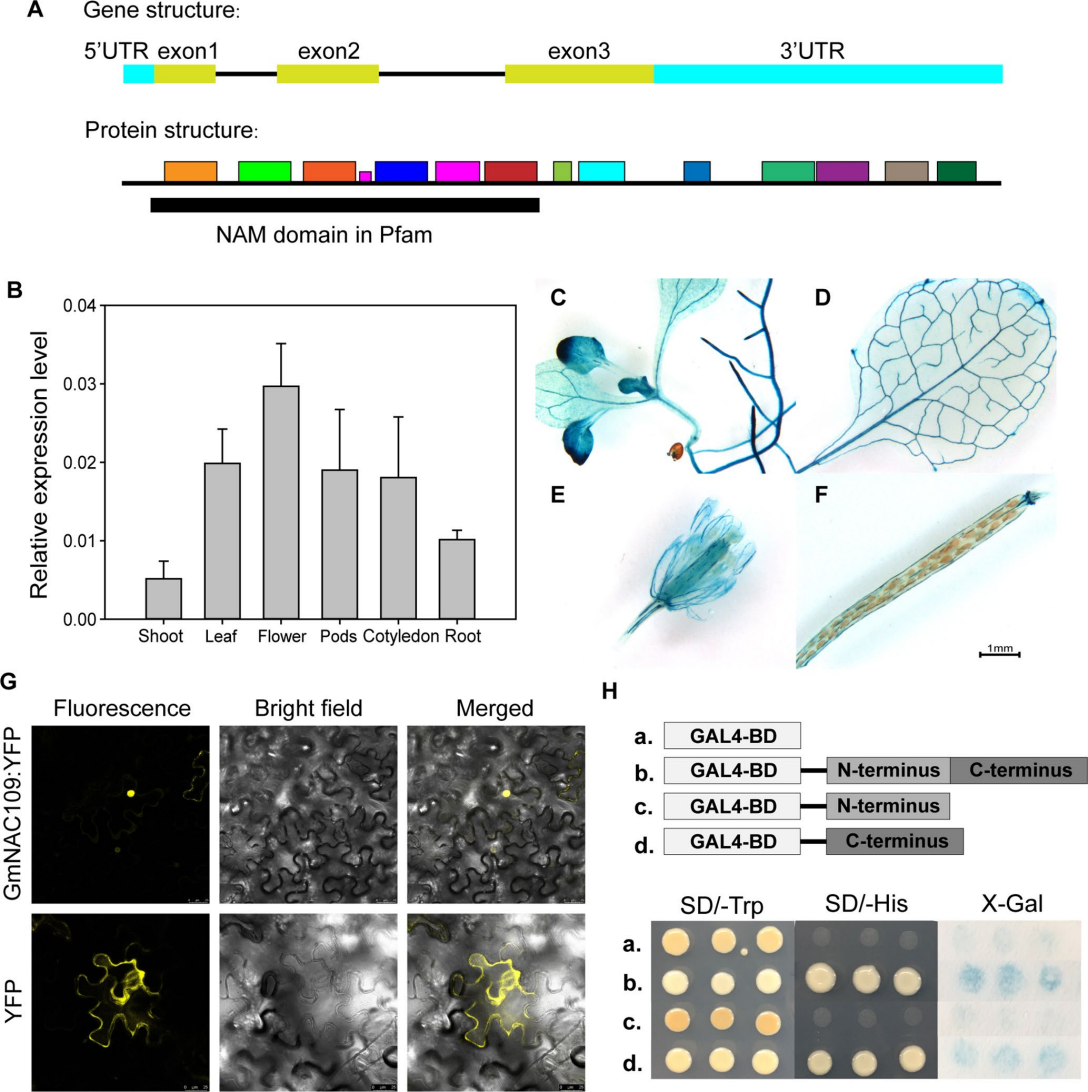
Tissue-specific expression, subcellular localization analysis, and transactivation assay of *GmNAC109*. **(A)** Gene and protein structures of *GmNAC109*. **(B)** qRT-PCR analysis of the relative expression level of *GmNAC109* in different tissues of soybean plants. **(C–F)** Gus staining assay of transgenic plants carrying GUS CDS sequence driven by *GmNAC109* promoter. **(G)** GmNAC109-YFP and empty YFP constructs were transiently expressed in tobacco leaves. The upper and lower rows show GmNAC109-YFP and YFP fluorescence, respectively. **(H)** (Upper) Schematic structure of full-length, N- and C-terminal fragments of GmNAC109 protein fused with GAL4BD. (Lower) Growth of the four transformants on SD/-Trp (first column) and SD/-His (second column) media. X-gal staining assay of colonies formed by the four transformants (third column). The pBKGT7 vector was used as a negative control.

The soybean NAC gene *GmNAC109* contains three exons, which are translated into a 281-amino-acid sequence with a conserved NAM domain at the N-terminus (**Figure 1A**). The protein motifs of GmNAC109 were predicted by MEME Suite (http://meme-suite.org/tools/meme) based on the *Arabidopsis* NAC superfamily gene *ATAF1* (AT1G01720) (**Figure 1A**). We initially investigated the expression pattern of *GmNAC109* in various tissues of soybean (cv. Williams 82) using qRT-PCR (**Figure 1B**). Under normal conditions, *GmNAC109* was expressed in shoot, leaf, root, cotyledon, flower, and pod tissues, and its expression was highest in flowers and lowest in shoots. The site of GUS expression of transgenic plants carrying the GUS CDS driven by *GmNAC109* promoter was examined by histochemical staining. *GmNAC109* was expressed throughout 7- to 10-day-old *Arabidopsis* seedlings including in roots, leaves, and hypocotyl (**Figures 1C–F**). Staining was strong in vascular tissues of leaves, roots, and the shoot apical meristem (**Figures 1C, D**), indicating that *GmNAC109* is highly expressed in these tissues. During the reproductive stage, the leaves, sepals, and calyces of silique were also stained in *Arabidopsis* T1 plants (**Figures 1E, F**).

### Subcellular Localization of GmNAC109

For detailed subcellular localization analysis of GmNAC109, its amino acid sequence was checked using two nuclear localization sequence database websites called NLSDB (https://rostlab.org/services/nlsdb) and iSPORT (http://ipsort.hgc.jp/); however, no signaling peptides were detected. To create a reporter construct, we fused YFP to the C-terminus of GmNAC109 (GmNAC109-YFP). The empty vector containing only *YFP* was used as a control. The constructs were infiltrated into tobacco leaves. While control YFP protein was mainly located in the cell membrane, the GmNAC109-YFP fusion protein accumulated in the nucleus (**Figure 1G**). This indicates that GmNAC109 is active in the nucleus.

### The C-terminal Domain of GmNAC109 Functions as a Transcriptional Activator

To investigate if GmNAC109 exhibits transcriptional activation activity, and if so, which domain is responsible for this activity, we divided GmNAC109 protein into two fragments: the N-terminal domain (amino acids 1–133) containing the NAM domain as predicted by NCBI Conserved Domain Database (CDD) and the C-terminal domain (amino acids 134–281). Full-length GmNAC109 as well as the N-terminal and C-terminal domains were fused to the C-terminus of the GAL4-binding domain (GAL4BD). The empty vector was used as a control. Yeast transformed with these four constructs was grown on SD selection medium. All four yeast transformants grew well on SD/-Trp medium (**Figure 1H**). Yeast transformed with GAL4BD fused to full-length GmNAC109 or the C-terminal domain survived on SD/-His medium, while yeast transformed with the empty vector and GAL4BD fused to the N-terminal domain did not (**Figure 1H**). Consistently, X-Gal only stained yeast colonies transformed with the fusion protein containing the C-terminal domain or full-length GmNAC109 in the colony lift colorimetric assay (**Figure 1H**). These results indicate that GmNAC109 has transcriptional activation activity and that its C-terminal domain plays an important role in this context.

### *GmNAC109* is Upregulated Under Abiotic Stress

**Figure 2:**
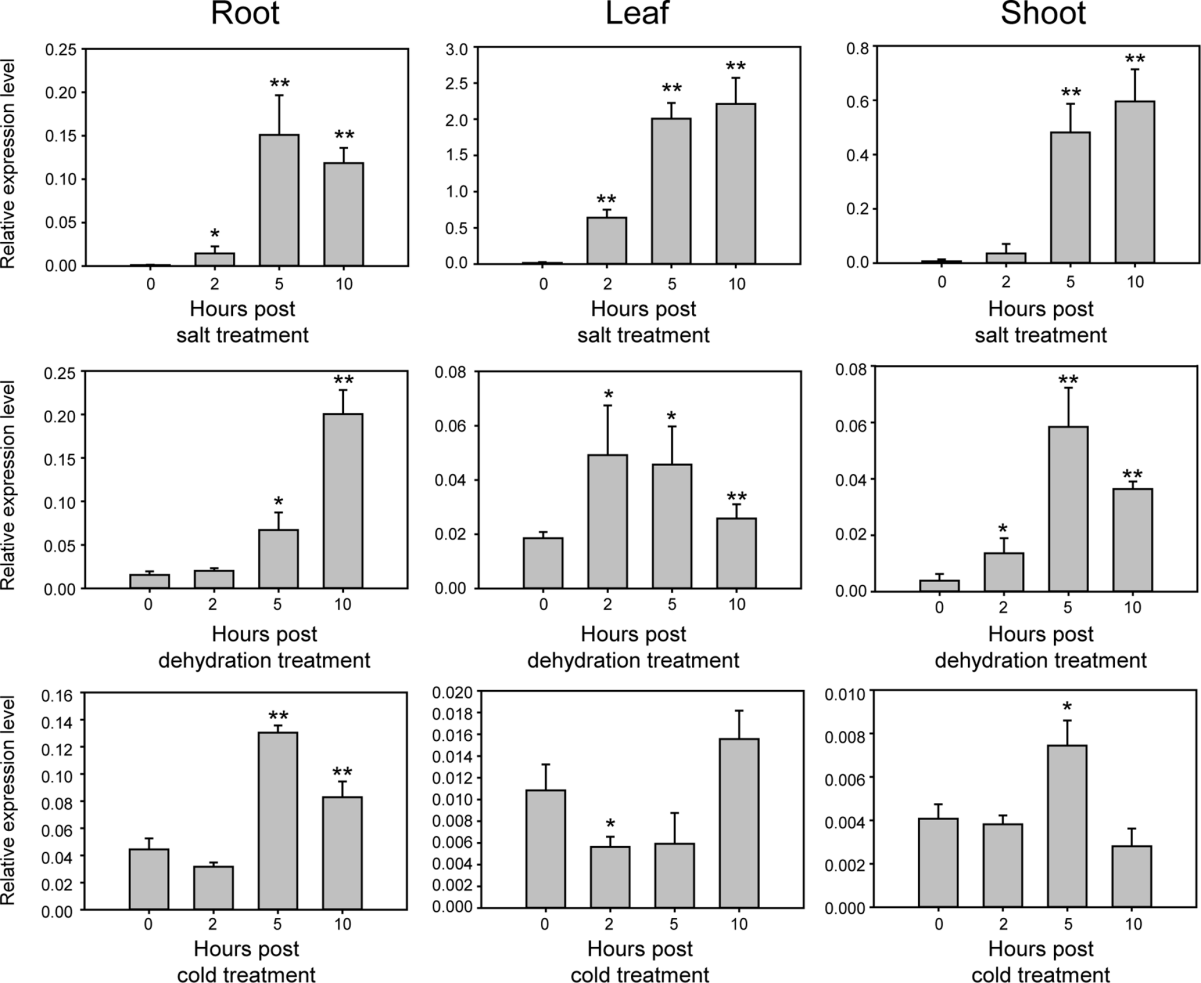
Expression of *GmNAC109* in various tissues of 14-day-old soybean seedlings under different stress treatments. Expression levels of *GmNAC109* were determined from soybean root, leaf and shoot tissues under salt, dehydration, and cold stresses using qPCR analysis. Soybean seedlings were treated with different stresses for 0, 2, 5, and 10 h. *P < 0.05; **P < 0.01.

Sequence analysis using BLAST showed that *GmNAC109* is homologous to the *Arabidopsis* NAC gene *ATAF1* (AT1G01720), which is responsible for resistance to abiotic stress (Wu et al., 2009), with a sequence identity of 72% and a similarity of 97% (data not shown). To examine whether *GmNAC109* responds to abiotic stress similar to *ATAF1*, we investigated the relative expression level of *GmNAC109* in various tissues of Williams 82 seedlings using qRT-PCR under three types of abiotic stress, namely, salt, dehydration, and cold (**Figure 2**). Under salt treatment, *GmNAC109* expression level was highly increased in leaves, roots, and shoots with treatment time. Dehydration stress significantly induced *GmNAC109* expression after 5 h of treatment in root and after 2 h in leaves and shoots. Under cold stress treatment, no significant change was observed in leaf tissues, but roots and shoots showed significant upregulation of *GmNAC109* after 5 h of treatment. These results demonstrate that salt, dehydration, and cold stresses highly induce *GmNAC109* expression in soybean plants.

### *GmNAC109* Overexpressed *Arabidopsis* Plants Showed Enhanced Lateral Root Formation and ABA Hypersensitivity

**Figure 3:**
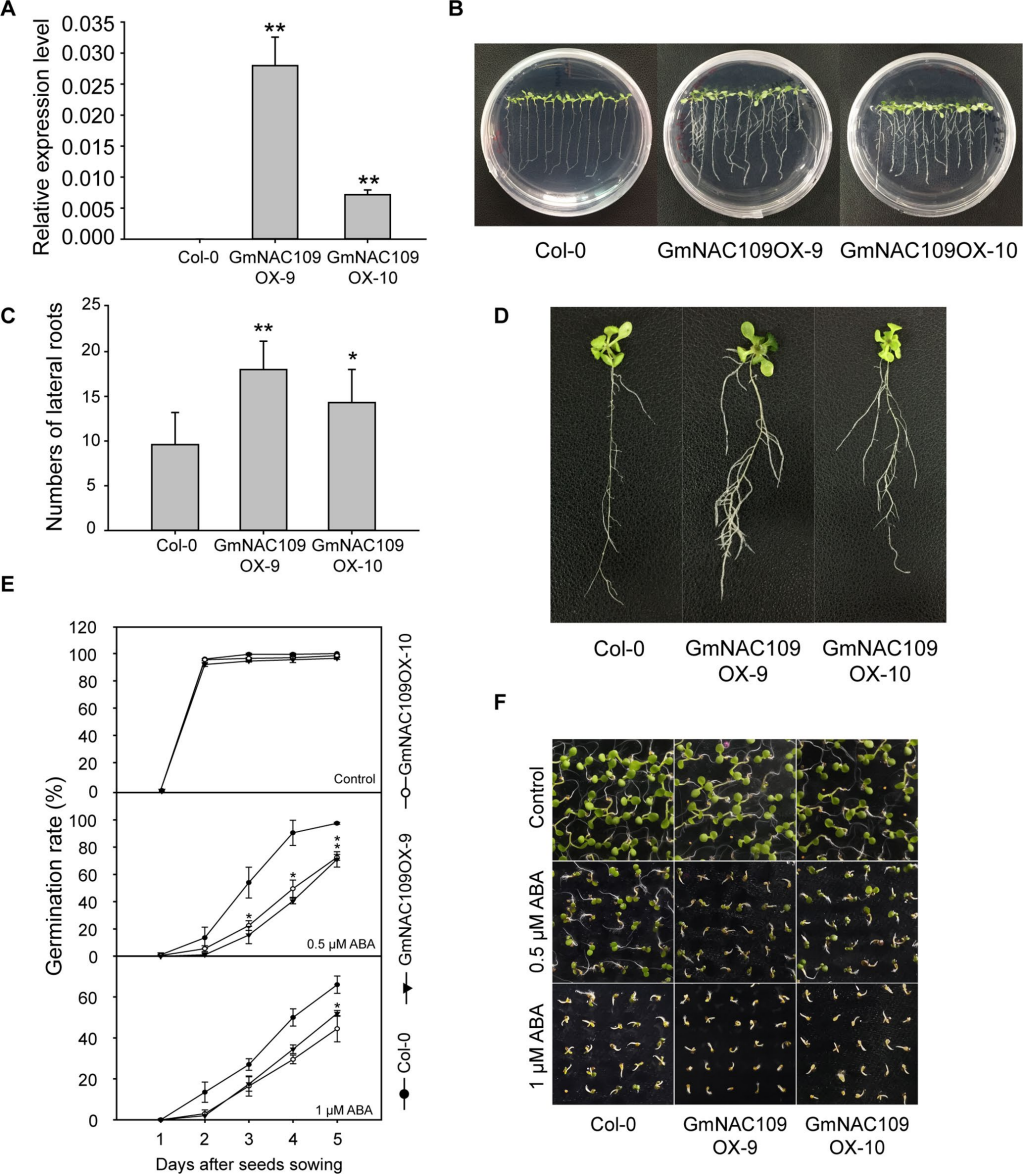
Phenotypic differences in lateral root formation and ABA sensitivity between Col-0 and *GmNAC109*-overexpressing transgenic lines. **(A)** Expression levels of *GmNAC109* in the two *Arabidopsis* transgenic lines GmNAC109OX-9 and GmNAC109OX-10. **(B)** Col-0 and T3 transgenic plants vertically grown on 1/2 MS medium. **(C)** Number of lateral roots in the Col-0, GmNAC109OX-9, and GmNAC109OX-10 lines. **(D)** Morphology of lateral roots in 3-week-old Col-0, GmNAC109OX-9, and GmNAC109OX-10 seedlings. **(E)** Seed germination rates of the Col-0, and GmNAC109OX lines in response to different ABA concentrations. **(F)** Photograph of seeds germinated on 1/2 MS medium containing 0, 0.5 or 1 μM ABA. *P < 0.05; **P < 0.01.

For functional characterization, *GmNAC109* was overexpressed in *Arabidopsis*. Among 20 *GmNAC109*-overexpressing transgenic *Arabidopsis* lines (GmNAC109OXs), 2 lines, 1 with the highest expression level and 1 with an intermediate expression level (**Figure 3A**), were selected for further analysis and designated GmNAC109OX-9 and GmNAC109OX-10, respectively. When grown on 1/2 MS medium, wild-type Col-0 and transgenic lines GmNAC109OX-9 and GmNAC109OX-10 seedlings exhibited obvious phenotypic differences in lateral roots (**Figures 3B, C**). Bulged nodes were counted as the number of lateral roots after 7 days of vertical cultivation. GmNAC109OX-9 and GmNAC109OX-10 seedlings formed significantly more lateral roots than Col-0 seedlings (**Figure 3D**). The number of lateral roots was almost 2- and 1.5-fold higher in GmNAC109OX-9 and GmNAC109OX-10 seedlings than in Col-0 seedlings, respectively. To investigate whether *GmNAC109* affects plant sensitivity to exogenous ABA, the seeds of Col-0 and transgenic lines were sowing on 1/2 MS medium supplemented with 0, 0.5, and 1 μM ABA. Germination rate was scored every 24 h after sowing. As shown in **Figures 3E, F**, the germination rates of GmNAC109OX-9 and GmNAC109OX-10 were significantly lower than Col-0 when treated with 0.5 and 1 μM ABA, but no difference was observed with 0 μM ABA. This indicates that the transgenic lines are hypersensitive to ABA compared to Col-0.

### Overexpression of *GmNAC109* Enhances Drought and Salt Stresses Tolerance in *Arabidopsis*

**Figure 4:**
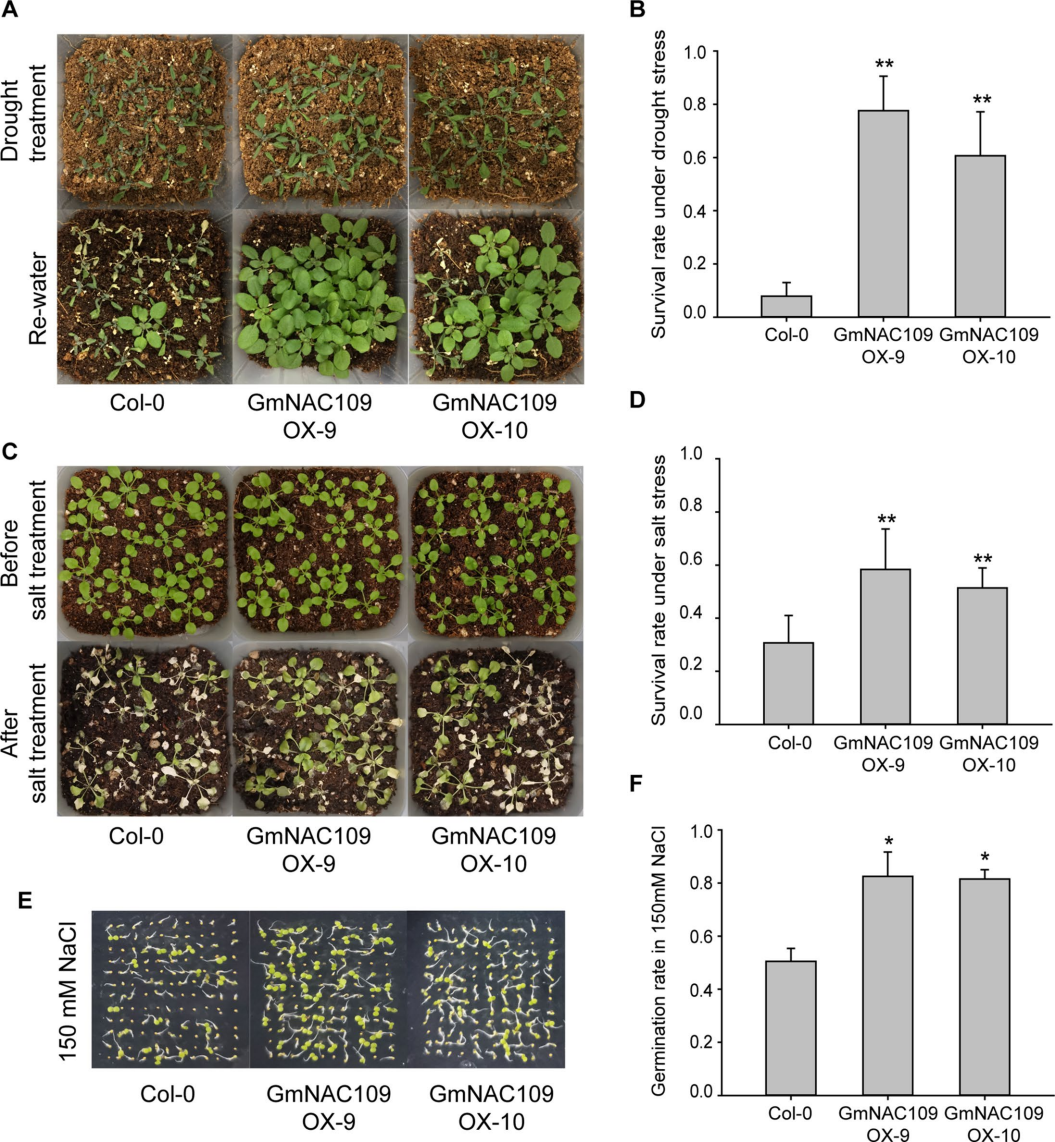
Response to drought and salt stress between the Col-0 and *GmNAC109*-overexpressing transgenic *Arabidopsis* lines. **(A)** Drought stress tolerance comparison. Photographs of the Col-0, GmNAC109OX-9, and GmNAC109OX-10 lines after 10 days without watering (upper) and after re-watering (lower). **(B)** Survival rate of the Col-0, GmNAC109OX-9, and GmNAC109OX-10 lines after drought stress and re-watering. **(C)** Salt stress tolerance comparison. Photographs of the Col-0, GmNAC109OX-9 and GmNAC109OX-10 lines before salt stress treatment (upper) and after 10 days of watering with 250 mM NaCl solution (lower). **(D)** Survival rate of the Col-0, GmNAC109OX-9, and GmNAC109OX-10 lines after salt treatment. **(E)** Photographs of seeds germinated on the medium containing150 mM NaCl. **(F)** Germination rate of the Col-0, GmNAC109OX-9, and GmNAC109OX-10 lines on the medium containing 150 mM NaCl. *P < 0.05; **P < 0.01.

To evaluate abiotic stress tolerance in the *GmNAC109*-overexpressing transgenic lines, GmNAC109OX-9, GmNAC109OX-10, and wild-type Col-0 seedlings were subjected to drought and salt stress treatments. After 10 days of drought stress, wilting of the Col-0 line was more severe than that of the transgenic lines, and significantly more GmNAC109OX-9 and GmNAC109OX-10 plants than Col-0 plants were revived after re-watering (**Figure 4A**). Only 8% (11 of 136 seedlings) of Col-0 plants were recovered, in comparison with 78% (98 of 126 seedlings) and 61% (82 of 134 seedlings) of GmNAC109OX-9 and GmNAC109OX-10 plants, respectively (**Figure 4B**). For salt stress treatment, the seedlings were treated with 250 mM NaCl solution for 10 days, and the transgenic lines survived better than Col-0 (**Figure 4C**). As shown in **Figure 4D**, GmNAC109OX-9 and GmNAC109OX-10 showed 58% (84 of 144 seedlings) and 51% (74 of 144 seedlings) survival rates, respectively, and the Col-0 survival rate was 32% (46 of 143 seedlings). Besides, we performed the germination assay of wild type and the transgenic lines on 150 mM NaCl-containing medium (**Figure 4E**). The germination rates of Col-0, GmNAC109OX-9, and GmNAC109OX-10 were 47%, 72%, and 54%, respectively. (**Figure 4F**) Overexpression of *GmNAC109* increased tolerance to drought and salt stresses in *Arabidopsis*, indicating that *GmNAC109* plays a role in stress responses.

### Overexpression of *GmNAC109* Increases Expression of Genes Related to Abiotic Stress Resistance and Lateral Root Formation

**Figure 5:**
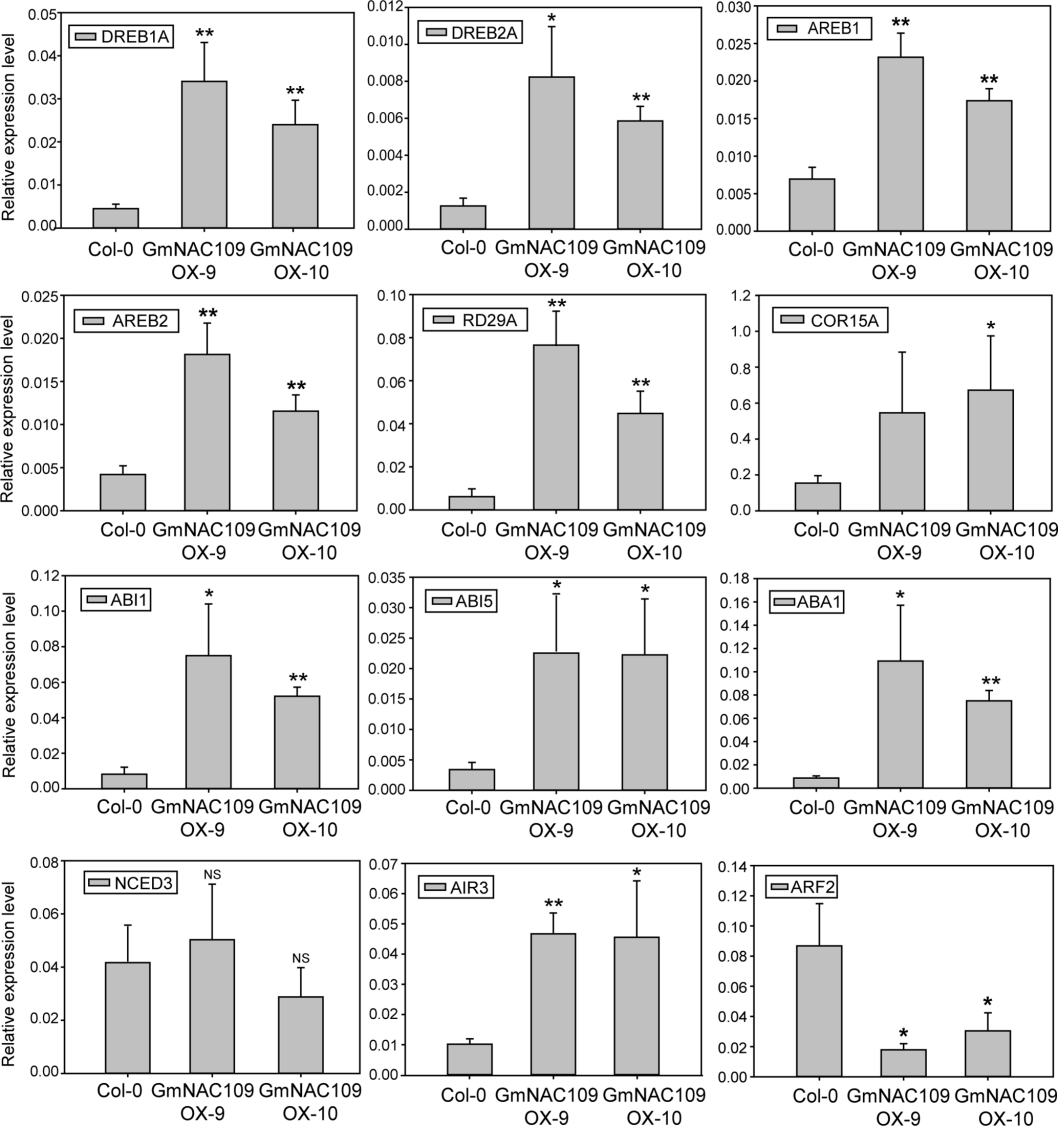
Relative expression of genes related to abiotic stress and lateral root formation in *GmNAC109*-overexpressing transgenic *Arabidopsis* lines. Expression levels of abiotic stress-related TFs (*DREB1A/2A* and *AREB1/2*), cold-responsive genes (*RDA29* and *COR15A*), ABA-related genes (*ABI1*, *ABI5*, *ABA1* and *NCED3*), and lateral root development-related genes (*AIR3* and *ARF2*) in the Col-0, GmNAC109OX-9, and GmNAC109OX-10 lines. Error bar represents the standard error for three independent replicates. *P < 0.05; **P < 0.01; NS, no significant differences.

To elucidate how *GmNAC109* increases stress tolerance and the number of lateral roots, we determined the expression levels of known stress and root development-related genes in Col-0, GmNAC109OX-9, and GmNAC109OX-10 plants. Six stress-related genes were selected, including four TF genes called *DREB1A* (*DROUGHT-RESPONSIVE ELEMENT-BINDING 1A*), *DREB2A*, *AREB1* (*ABSCISIC ACID–RESPONSIVE ELEMENT BINDING PROTEIN 1*), and *AREB2*, and two cold-responsive genes called *RD29A* (*RESPONSIVE TO DESICCATION 29A*) and *COR15A* (*COLD REGULATED 15A*). Expression of all these genes was higher in the transgenic lines than in the Col-0 line (**Figure 5**). Moreover, expression of these genes was higher in the GmNAC109OX-9 line, in which the extent of *GmNAC109* overexpression was greater, than in the GmNAC109OX-10 line (**Figure 3A**). These results support the finding that GmNAC109OX-9 plants were more tolerant of drought than GmNAC109OX-10 plants.

Since the transgenic lines were hypersensitive to ABA (**Figures 3E, F**), we investigated the expression level of four ABA-related genes. *ABI1* (*ABA INSENSITIVE 1*) and *ABI5*, involved in ABA responsiveness, were highly upregulated in GmNAC109OX-9 and GmNAC109OX-10. However, two genes related with ABA biosynthesis showed different expression levels; *ABA1* (*ABSCISIC-ACID 1*) encoding zeaxanthin epoxidase in the ABA biosynthetic pathway was upregulated in two *GmNAC109*-overexpressing *Arabidopsis* lines, while *NCED3* (*NINE-CIS-EPOXYCAROTENOID DIOXYGENASE 3*), a key enzyme gene in the ABA biosynthetic pathway, had no differential expression between wild type and the transgenic lines. To check whether elevated expression of the ABA-responsive genes results from increase of endogenous ABA amount or not, we measured the ABA contents from the seedlings of Col-0, GmNAC109OX-9, and GmNAC109OX-10 under control and drought treatment. No significant difference in the ABA contents was observed between Col-0 and the transgenic lines (**Supplementary Figure S1**). In spite of induction of *ABA1*, overexpression of *GmNAC109* is supposed to increase ABA responsiveness rather than ABA biosynthesis itself.

To assess lateral root development, expression of two auxin-responsive genes called *AIR3* (*AUXIN-INDUCED IN ROOT CULTURES 3*) and *ARF2* (*AUXIN RESPONSE FACTOR 2*) was analyzed. *AIR3* acts downstream of *NAC1*, which controls lateral root development in *Arabidopsis* (Xie et al., 2000). Expression of *AIR3* was higher in both *GmNAC109*-overexpressing transgenic lines than in the Col-0 line (**Figure 5**). Meanwhile, expression of *ARF2* was lower in the GmNAC109OX-9 and GmNAC109OX-10 lines than in the Col-0 line and was lower in the GmNAC109OX-9 line than in the GmNAC109OX-10 line. Based on these results, *GmNAC109* increases abiotic stress tolerance by affecting the expression levels of these stress-related and ABA-responsive genes. In addition, *GmNAC109* appears to increase lateral root growth by upregulating *AIR3* and downregulating *ARF2*.

## Discussion

NAC genes are involved in various signaling pathways underlying plant biotic and abiotic stress responses as well as developmental processes. Although several studies have investigated the NAC family in plants, little is known about the functions of NAC genes in soybean. Genome-wide analysis of the NAC family has been conducted in soybean (Le et al., 2011; Hussain et al., 2017; Melo et al., 2018); however, the function of a single *GmNAC* gene has not been studied. In this study, we characterized the features and functions of soybean *GmNAC109* using transgenic *Arabidopsis* plants. To date, 180 members of NAC family have been identified in the soybean genome, which can be divided into 15 phylogenetic subfamilies (Melo et al., 2018). *GmNAC109* belongs to the SNAC-A (ATAF) subfamily. NACs contain a highly conserved N-terminal DNA-binding NAC domain and a variable transcription-regulating C-terminal domain (Ooka et al., 2003). The current study demonstrated that *GmNAC109* was upregulated in response to various types of abiotic stresses, including high salt, dehydration, and cold, and showed tissue-specific expression in roots and leaves (**Figure 2**). This implies that *GmNAC109* has diverse roles in response to environmental signals at different developmental stages.

In this study, the survival rates of *GmNAC109*-overexpressing *Arabidopsis* transgenic lines were significantly higher than those of wild-type plants under high-salt and drought conditions (**Figures 3E, F**). Overexpression of *GmNAC109* altered the expression levels of stress-responsive TFs and ABA-responsive genes. ABA is a phytohormone that accumulates under osmotic stress caused by drought and high salt, and ABA and ABA-responsive genes play an important role in stress responses and tolerance (Tuteja, 2007). In response to drought, ABA-responsive genes are regulated by interactions of multiple TFs. DREB/CBF TFs interact physically with ABA-responsive element (ABRE)-binding protein/ABRE-binding factor (AREB/ABF) TFs (Lee et al., 2010). NAC genes are also involved in the ABA biosynthesis and signaling pathways (Fujita et al., 2004; Jensen et al., 2013). The *Arabidopsis* SNAC TF *ATAF1* binds to the promoter of *NCED3* to regulate ABA biosynthesis (Jensen et al., 2013). However, *GmNAC109* did not induce *NCED3* in our transgenic lines (**Figure 4**). Moreover, Col-0 and the transgenic lines exhibited no significant difference in endogenous ABA contents (**Supplementary Figure S1**). *ANAC096* cooperates with AREB/ABF factors in response to osmotic stress (Xu et al., 2013). In our study, *GmNAC109* increased expression of *DREB1A/2A* and *AREB1/2*, which together can activate ABA-responsive genes. *GmNAC109* also upregulated two ABA-responsive genes *ABI1* and *ABI5* (**Figure 4**) and increased ABA sensitivity (**Figure 3**). Thus, further studies are necessary to identify how *GmNAC109* functions in ABA signaling pathway to enhance the tolerance to abiotic stress.

The *GmNAC109*-overexpressing transgenic *Arabidopsis* lines had significantly more lateral roots than the Col-0 line, while root length did not significantly differ (**Figures 3C, D**). This change in root structure may affect tolerance to high salt and drought because lateral root development is important in plants adapting to the environment and coping with abiotic stresses such as drought. Auxin plays an integral role in regulation of lateral root formation (Lavenus et al., 2013), and NACs are also reportedly involved in this process (Xie et al., 2000). *Arabidopsis NAC1* acts downstream of the auxin receptor *Transport Inhibitor Response 1* (*TIR1*) and activates expression of two downstream auxin-responsive genes called *AIR3* and *DBP* to promote lateral root development (Xie et al., 2000). *NAC1* is also regulated by *miR164*, which is induced by auxin, *via* a signaling pathway that is dependent on *Auxin Response Element 1* (*AXR1*), *AXR2*, and *TIR1* (Guo et al., 2005). The two transgenic lines, GmNAC109OX-9 and GmNAC109OX-10, exhibited upregulation of *AIR3* and downregulation of *ARF2* in comparison with the Col-0 line (**Figure 4**). *ARF2* was reported to function as upstream of NACs, and *arf2* mutant lines exhibit impaired lateral root formation (Okushima et al., 2005). In contrast with this previous study, we found that *GmNAC109* negatively regulated expression of *ARF2* and promoted lateral root formation. This indicates that either *GmNAC109* acts as an upstream regulator of *ARF2* or they mutually affect expression of each other. Therefore, our results indicate that *GmNAC109* is involved in the auxin pathway and promotes lateral root development by altering *AIR3* and *ARF2* expression; however, regulation of these genetic modules in signaling pathways for lateral root development is complicated and requires further study.

In *Arabidopsis*, overexpression of the *GmNAC109* homolog *ATAF1* confers drought tolerance and causes dwarfism (Wu et al., 2009). This indicates that *ATAF1* is active in the SAM. However, although *GmNAC109* was highly expressed in the SAM of soybean, dwarfism was not observed in *GmNAC109*-overexpressing lines. This functional difference between the closest orthologous NAC genes of soybean and *Arabidopsis* may be due to the presence of many paralogs caused by polyploidization in soybean (Schmutz et al., 2010). There are two other *ATAF1* homologs in soybean, called *GmNAC11* and *GmNAC20*, which are also induced by drought stress and regulate lateral root development (Hao et al., 2011), indicating that NACs have functional redundancy in soybean. Thus, changes in expression of a single gene may not have the same severe phenotypic effect in soybean as it does in *Arabidopsis*.

In conclusion, we characterized the function of *GmNAC109*, a member of the soybean NAC family, in response to abiotic stress using *GmNAC109*-overexpressing transgenic *Arabidopsis* lines. The survival rates of these lines were significantly higher than that of the control line under drought and salt stress. We also found that *GmNAC109* has functional redundancy with some NAC genes. The specific and redundant functions of individual members of the large NAC family in soybean must be studied further. This will provide comprehensive insight into stress defense responses and provide a basis for development of soybean lines with improved tolerance to abiotic stresses *via* genetic manipulation.

## Data Availability

No datasets were generated or analyzed for this study.

## Author Contributions

XY, MYK, and JH conceived and designed the research. XY conducted the experiments and analyzed the data. XY and MYK wrote the manuscript. JH and S-HL revised the manuscript. S-HL supervised the project. All authors read and approved the manuscript.

## Funding

This work was supported by the Next Generation BioGreen 21 Program (Code No. PJ01322401), Rural Development Administration, Republic of Korea.

## Conflict of Interest Statement

The authors declare that the research was conducted in the absence of any commercial or financial relationships that could be construed as a potential conflict of interest.
