# Supplementary material for: Overexpression of the Soybean NAC Gene GmNAC109 Increases Lateral Root Formation and Abiotic Stress Tolerance in Transgenic Arabidopsis Plants
Source: Front Plant Sci. 2019 Aug 16;10:1036. doi: 10.3389/fpls.2019.01036 (PMC6707213; doi:10.3389/fpls.2019.01036)
Supplement: Supplementary file 1 [file Table_1.docx]

**Table S1.** Primers used for vector construction

| Primer name | Primer Sequence (5’-3’) |
| --- | --- |
| NAC109_nonstop_attb_F | GGGGACAAGTTTGTACAAAAAAGCAGGCTATGGCCACTACAACACAACTT |
| NAC109_nonstop_attb_R | GGGGACCACTTTGTACAAGAAAGCTGGGTCATTGCAGAAGGACTTGGAGA |
| NAC109_FL_NcoI_F | CATGCCATGGCAATGGCCACTACAACACAACT |
| NAC109_FL_EcoR1_R | CGGAATTCTTAATTGCAGAAGGACTTGGAGA |
| NAC109_N_EcoR1_R | CGGAATTCTTAAAGACGATACTCGTGCATGA |
| NAC109_C_Nco1_F | CATGCCATGGCAGCAGACGTGGATCGTTCCGT |
| pNAC109_R | TGTTGAGAATTGAGAAAGAAAAT |
| pNAC109-2kb_F | CACCGCTAGCTGCCATTCTGTTGA |
| GmNAC109-F | CACCATGGCCACTACAACACAAC |
| GmNAC109-R | TTAATTGCAGAAGGACTTGGAGA |

**Table S1.** Primers used for qRT-PCR

| Primer name | Primer sequence (5’ -3’) |
| --- | --- |
| ACTIN11_F | CGGTGGTTCTATCTTGGCATC |
| ACTIN11_R | GTCTTTCGCTTCAATAACCCTA |
| EF1A_F | GACCTTCTTCGTTTCTCGCA |
| EF1A_R | CGAACCTCTCAATCACACGC |
| GmNAC109_qPCR_F | ACATATCGCGGTTCCCATAA |
| GmNAC109_qPCR_R | GAACCGTTCGGGTACTTGC |
| ERF5_F | TTGAAGACGGAACAGAGC |
| ERF5_R | AGGAGATAACGGCGACAG |
| RD29A_F | GGAAGAGTCGGCTGTTTCAG |
| RD29A_R | CAATCTCCGGTACTCCTCCA |
| DREB1A_F | GTTTCCTCAGGCGGTGATTA |
| DREB1A_R | TCTCCGACGAACTCCTCTGT |
| DREB2A_F | GTGACCTAAATGGCGACGAT |
| DREB2A_R | GCGGATCAAAACCACTTTGT |
| COR15A_F | TGATCTACGCCGCTAAAGGT |
| COR15A_R | CGCTTTCTCACCATCTGCTA |
| ABA1_F | ACTTGTTACACGGGGATTGC |
| ABA1_R | CCACCAACATCCGAAGAAAC |
| ABI1_F | TGGTCGGTTTGATCCTCAAT |
| ABI1_R | TAGCTATCTCCTCCGCCAAA |
| ABI5_F | GAGACTGCGGCTAGACAACC |
| ABI5_R | GGTTCGGGTTTGGATTAGGT |
| AIR3-F | TCGCTGCTCATCTAGACCAC |
| AIR3-R | CCATGATCGAGTCGTGTGAA |
| ERF5_F | TTGAAGACGGAACAGAGC |
| ERF5_R | AGGAGATAACGGCGACAG |
| AXR1_F | GGAACAATTTCATGGTCGATGC |
| AXR1_R | TCTCCTCAATAAACTTGGCGT |
| AXR3_F | GTTCCTTGGCCAATGTTCGT |
| AXR3_R | TCAAGCTCTGCTCTTGCACT |
| ARF2_F | ATCAGATGCAAAGGACGCCA |
| ARF2_R | GACCCCCATCCTCGGCTT |
| NCED3_F | GCTGCGGTTTCTGGGAGAT |
| NCED3_R | GACACGACTGGCCATAGGTA |


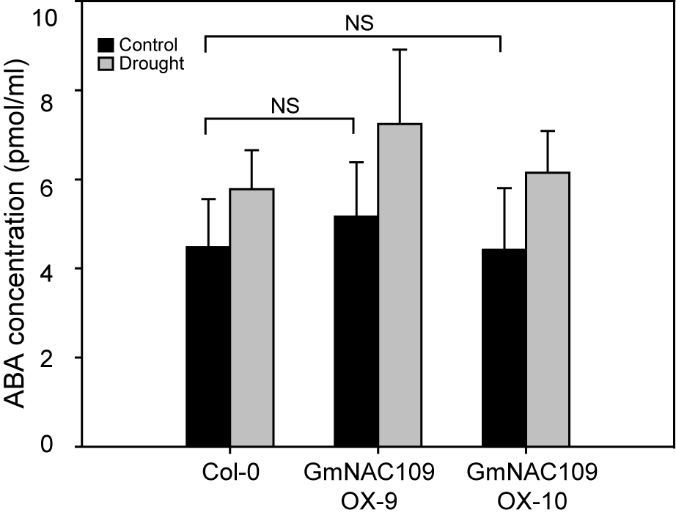


**Figure S1** ABA content of Col-0 and *GmNAC109*-overexpressing *Arabidopsis* transgenic lines under normal condition and drought stress. Drought stress treated seedlings were grown on 1/2 MS medium containing 75 mM mannitol. NS, no significant differences
